# Moxibustion ameliorates abnormal subchondral bone remodeling by promoting ACSL1-mediated autophagy to degrade NLRP3 in osteoarthritis

**DOI:** 10.1186/s13020-025-01182-2

**Published:** 2025-08-11

**Authors:** Xuelan Chen, Liping Fu, Yu Huang, Qing Liao, Binhua Zou, Lixia Yuan, Gang Liu

**Affiliations:** 1 Department of Rehabilitation Medicine, Nanfang Hospital, Southern Medical University, Guangzhou, 510515 Guangdong China; 2 School of Traditional Chinese Medicine, Southern Medical University, Guangzhou, 510515 Guangdong China

**Keywords:** Moxibustion, Osteoarthritis, Subchondral bone, ACSL1, Autophagy, NLRP3

## Abstract

**Background:**

Osteoarthritis (OA) is a joint disorder that is characterized, among other features, by abnormal subchondral bone remodeling. Moxibustion, a traditional Chinese medicine treatment, has a long history in the clinical treatment of osteoarthritis and has demonstrated significant efficacy. However, the impact mechanisms of moxibustion on subchondral bone in osteoarthritis have yet to be elucidated.

**Purpose:**

This study investigated the specific effects and mechanisms of moxibustion on abnormal subchondral bone remodeling in OA.

**Methods:**

Anterior cruciate ligament transection (ACLT) surgery was performed on mice to establish an OA model, and moxibustion intervention for 4 weeks. The effects of moxibustion on knee osteoarthritis symptoms and walking ability were assessed by knee joint diameter measurement, von Frey test and footprint analysis. Micro-CT, TEM, immunofluorescence staining, and western blot were used to detect the contact between autophagy–lysosomal pathway and NLRP3 inflammasome in subchondral bone remodeling. Subsequently, proteomic analysis was performed on mouse subchondral bone.

**Results:**

We first discovered that moxibustion intervention effectively reduced inflammation in the subchondral bone, thereby balancing the activities of osteoblasts and osteoclasts. Moxibustion, with its warming and medicinal properties, significantly alleviated pain and swelling and enhanced walking ability in OA mice. The findings also suggested that moxibustion counteracted subchondral bone imbalance by inhibiting the activation of the NLRP3 inflammasome through increased autolysosome levels. Proteomic analysis and experimental validation revealed that moxibustion promoted ACSL1 expression to regulate autophagy in OA subchondral bone.

**Conclusion:**

Our study elucidated the molecular mechanism by which moxibustion improved the inflammatory environment and abnormal subchondral bone remodeling in OA mice by activating ACSL1-mediated autophagy, providing the basis and new insights for  advancing moxibustion therapy in OA.

**Supplementary Information:**

The online version contains supplementary material available at 10.1186/s13020-025-01182-2.

## Introduction

Osteoarthritis (OA) is a chronic, progressive and disabling disease characterized by total joint disorder, including hyaline cartilage erosion, abnormal subchondral bone remodeling, synovial hyperplasia, as well as increased vascularity [1, 2]. Recent studies have shown that locally enhanced subchondral bone remodeling, along with microstructural and biomechanical alterations, negatively impacts the overlying cartilage [3–5]. Risk factors like obesity [6], ligament injury [7] and aging-related muscle weakness [8] increase the mechanical loading of weight-bearing joints, resulting in the disruption of subchondral bone homeostasis and the development of lesions. Abnormal subchondral bone remodeling in OA is closely associated with cellular metabolism and vascular proliferation [9, 10]. Therefore, studying the causes, identifying therapeutic targets, and advancing pharmacological development for abnormal subchondral bone remodeling is critical for improving the prognosis of OA patients.

Increased expression of pro-inflammatory cytokines in subchondral bone is believed to be connected with the onset and progression of structural changes in OA joints, particularly interleukin (IL)−1β, which alters osteoblast phenotype [11, 12]. The NOD-like receptor thermal protein domain associated protein 3 (NLRP3) inflammasome is a molecular platform for activating Caspase-1, a cysteine protease that cleaves and stimulates the release of pro-inflammatory cytokines IL-1β and IL-18 [13, 14]. Activation of NLRP3, as an endogenous danger signal, induces subchondral bone inflammation and accelerates bone remodeling [15, 16]. Nevertheless, growing evidence suggests that autophagy functions as a negative regulator of NLRP3 activation [17, 18]. The fusion of autophagosomes and lysosomes produces autolysosomes that aim to degrade harmful compounds and regulate inflammation [19, 20]. Therefore, targeting the NLRP3 inflammasome and autophagy–lysosome dysfunction in subchondral bone may represent a promising therapeutic strategy for OA.

Moxibustion is a traditional Chinese medicine therapy, first recorded in “Huangdi Neijing” more than 2000 years ago. This treatment utilizes the heat, infrared radiation and volatile oils generated by burning Artemisia argyi to directly stimulate the acupuncture points, achieving body regulation and disease prevention [21, 22]. Several studies and clinical applications have indicated that moxibustion is effective in treating OA [23, 24]. Flavonoids and polysaccharide components in Artemisia argyi exhibit significant anti-inflammatory and anti-stress characteristics [25]. It has been demonstrated that moxibustion at Zusanli (ST36) enhances immune function and alleviates joint injury [26]. The combination of Zusanli (ST36) and Shenshu (BL23) can alleviate inflammation and cartilage destruction in OA, as evidenced by studies [27]. Our previous research found that moxibustion attenuates osteosclerosis in the OA subchondral bone [21]. However, the exact mechanism remains unclear.

In this study, we explored for the first time the specific mechanism by which moxibustion improves abnormal subchondral bone remodeling in OA. It was shown that moxibustion could maintain bone remodeling homeostasis by activating ACSL1 to regulate autolysosomal levels and diminish the release of inflammatory factors in OA subchondral bone.

## Materials and methods

### Animals

Male C57BL/6J mice (age: 8 weeks old) were obtained from the Animal Center of Southern Medical University, Guangzhou, China. The mice were housed in a 12-h light/dark cycle, provided with regular food and water, and maintained at a constant temperature of 24 ± 2 °C. The use of animals and experimental procedures were approved by Nanfang Hospital Animal Ethic Committee.

According to previous reports, anterior cruciate ligament transection (ACLT) was performed to create surgical joint instability and establish the OA model [21]. After 7 days of adaptive feeding, all mice were randomly divided into three groups: (1) the sham surgery group (SHAM); (2) the ACLT induced OA group (ACLT); (3) the ACLT with moxibustion group (ACLT + M).

Moxibustion treatment commenced after ACLT surgery and continued for 28 days, with each session lasting 20 min. Following a previously published method [28], the moxa sticks (Nanyang Grass Jelly Pharmaceutical Co., Ltd, China) with a 0.5 cm diameter were placed 2–3 cm above the Zusanli (ST36) and Shenshu (BL23), which were identified according to the acupuncture point atlas. A thermometer was used throughout the procedure to maintain the skin surface temperature of the acupoints at 43 °C–46 °C.

### Swelling and strength measurements

To evaluate knee swelling, the diameter of each mouse's right knee joint was measured using a digital caliper before and weekly after ACLT surgery. Thigh circumference was measured using a flexible measuring tape at the end of therapy period. Additionally, limb grasp strength and hind paw strength were assessed using a grip strength meter. Each mouse from different groups was measured at least three times.

### Von Frey testing

The tactile sensitivity of hind paws in mice was assessed using a dynamic plantar aesthesiometer (Ugo Basile, Shanghai). The animals were allowed 20 min to adapt after being put in elevated plexiglas chambers with metal mesh bottoms. Following the cessation of the grooming or exploratory behavior, a straight metal filament (0.5 mm in diameter) was pressed perpendicular to the hind paw’s plantar surface (avoid the toe pads). The metal filament exerted an increasing upward force when it touched the plantar surface, which was stopped by the hind paw shaking, flicking, or withdrawing. Measurements were taken in triplicate at 10-min intervals, and the average value was recorded as the paw withdrawal threshold.

### Gait analysis

At the end of the treatment, the gait of the mice was assessed using footprint analysis. The left hind paws were dipped in blue ink, and the right hind paws with red ink. Mice were allowed to freely move back and forth on a pathway that was 50 cm long and 10 cm wide. The footprints on the walkway were recorded on paper. Each mouse was evaluated at least three times during the procedure, which took place in a rather darkroom setting. Lastly, the footprint parameters were measured and analyzed.

### Micro-computed tomography analysis

Utilizing a Bruker Micro-CT SkyScan 1276 system with a voltage of 85 kV and 200 mA current, a voxel size of 6 μm, and medium resolution, the knee joint specimens fixed with 4% paraformaldehyde were scanned. Osteoid development and subchondral osteosclerosis were observed in detail in the sagittal and coronal planes. 3D reconstruction and data analysis were then performed using DataViewer 1.5.1.9, CTAnv1.15.4, and CTvol2.2.3.0 software.

### Transmission electron microscopy (TEM)

Right knee subchondral bone samples were fixed with 2.5% glutaraldehyde, decalcified and subjected to acetone gradient dehydration. The samples were then immersed in a graded mixture of acetone and embedding agent for gradient infiltration. Following this, the tissue blocks were embedded and dried to form solid blocks. Sections were sectioned at 50 nm thickness with an ultramicrotome, attached to copper grids, and stained with uranyl acetate and lead citrate. Transmission electron microscopy (FEI Tecnai G2 Spirit) was used for observation.

### Histological analysis

Following a 25-day period of continuous decalcification, the joint tissues were embedded in paraffin and cut into 4-µm-thick sections. In accordance with the kit’s operating instructions, dewaxing was followed by Safranin O-Fast Green staining (Sigma-Aldrich), hematoxylin and eosin (H&E) staining and tartrate-resistant acid phosphatase (TRAP) staining (Servicebio). The pathological features of cartilage were analyzed using the Mankin’s scoring system and Osteoarthritis Research Society International (OARSI) scoring system [29].

In addition, Safranin O-Fast Green staining was used to assess medial tibial plateau osteophyte formation, including osteophyte size and osteophyte maturity. The osteoid size was determined by measuring the medial and lateral widths of the widest portion of the osteoid [30]. Osteoid maturity ratings varied from 0 to 3: 0 indicates no osteoid, 1 indicates predominantly cartilaginous osteoid, 2 indicates a mixture of cartilage and bone, and 3 indicates predominantly bony structures [31].

### Immunohistochemical analysis

Knee paraffin slices was incubated with primary antibody against RUNX2 (ZENBIO, R25634; 1:200), IL-1β (Abmart, P50520-1R1F; 1:50), IL-18 (Abmart, M027287F; 1:150), and Beclin 1 (Abmart, T55092F; 1:150) at 4 °C overnight and then incubated with goat anti-rabbit/mouse secondary antibodies (ZENBIO, 511203/511103; 1:200) for 1 h at room temperature. Exposed to diaminobenzidine (DAB) peroxidase substrate (Zhongshan Golden Bridge, ZLI-9017), the slices counterstained with hematoxylin, and dehydrated through an ethanol gradient. The images were captured using a slide scanning system SQS40P (Shengqiang Technology Co., Shenzhen).

### Immunofluorescence analysis

Primary antibody against NLRP3 (Proteintech, 68102-1-I; 1:80), Caspase-1 (Abmart, M025280F; 1:150), SQSTM1 (Abmart, T55546F; 1:200), LAMP1 (Proteintech, 21997-1-AP; 1:200), LC3B (Proteintech, 18725-1-AP; 1:200), ATG5 (Abmart, T55766F; 1:100) and PPARγ (ZENBIO, 340844; 1:200) were added to the slices at 4 °C for over 12 h. After being incubated with anti-rabbit or anti-mouse fluorescent secondary antibodies (Abbkine, 1:200) at room temperature for 1 h, all sections were stained with DAPI (LEAGENE; DA0001) and observed under the fluorescence microscope.

### Western blotting

The subchondral bone or cartilage tissue was ground into powdered granules using liquid nitrogen and treated with RIPA lysis buffer (Fdbio Science, FD009) supplemented with Protease Inhibitor Cocktail (CWbio, CW2200S). Following isolation, the proteins were transferred onto a polyvinylidene fluoride membrane and then incubated with the relevant primary antibody against β-actin (Proteintech, 66009-1-Ig; 1:1000), MMP13 (Proteintech, 18165-1-A; 1:1000), ADAMTS5 (Affinity, DF13268, 1:1000), COL2A1 (Servicebio, GB11021; 1:1000), OSX (Abcam, ab209484; 1:1000), RUNX2 (ZENBIO, R25634; 1:1000), CTSK (Santa cruze, SC-48353; 1:500), IL-1β (Abmart, P50520-1R1F; 1:1000), IL18 (Abmart, P50519-2R2F; 1:1000), Beclin 1 (Abmart, T55092F; 1:1000), NLRP3 (Proteintech, 68102-1-I; 1:750), total and cleaved caspase1 (Abmart, M025280F; 1:1000), SQSTM1 (Abmart, T55546F; 1:1000), LAMP1 (Proteintech, 21997-1-AP; 1: 1000), LC3B (Sigma, L7543; 1:2000), ATG5 (Abmart, T55766F; 1:1000), PPARγ (ZENBIO, 340844; 1:1000), ACSL1 (HUABIO, HA601112, 1:1000), mTOR (Abmart, T55306F; 1:1000), Phospho-mTOR (Abmart, T56571F; 1:500), ULK1 (Affinity, DF7588; 1:1000), Phospho-ULK1 (Affinity, AF4387; 1:500), AMPKα (Abmart, T55326F; 1:1000), Phospho-AMPKα (Abmart, T55608F; 1:1000), overnight at 4 °C. The secondary antibody (ZENBIO, 511103/511203; 1:200) was incubated with membranes for 1 h at 25 °C.

### Proteomics assay

Subchondral bone tissue samples were ground rapidly in liquid nitrogen. Approximately 15 mg of the homogenate was lysed with 150 μL of lysis buffer (FDbio Science, FD009). Following reduction with 10 mM dithiothreitol and alkylation with iodoacetamide at 25 °C for 30 min under light protection, the protein solution samples were digested and processed using MS-grade trypsin in ultrafiltration centrifuge tubes. Lastly, trypsin was added to the protein supernatants and incubated overnight at 37 °C. After enzymatic lysis, the peptides were vacuum-freeze-dried and desalted using Strata X C18 (Phenomenex).

High-pH reverse-phase chromatography was performed using an Agilent 300 Extend C18 column to fractionate the samples. A gradient of increasing acetonitrile concentration (pH = 9.6) from 8 to 32%, was used to separate the mix samples. After being gathered along the gradient, the fractions were dried for further LC–MS/MS analysis. Data collection for separated peptides was accomplished using a nano-spray-ionization (NSI) ion source and a Q Exactive HF-X MS. For MS analysis, the profile data was captured using data-dependent acquisition (DDA) at a resolution of 120,000 within the m/z range of 400–1600.

### Mass spectrometry data analysis

MaxQuant software (version 1.6.2.3), Perseus and Chiplot were used to analyze bioinformatics data. A fold-change ratio of > 2 or < 0.5 and p-value < 0.05 were used as the cutoffs for filtering differentially expressed proteins (DEPS). With hierarchical clustering, expression data were categorized based on protein level. Information details were collected from UniProtKB, Kyoto Encyclopedia of Genes and Genomes (KEGG) and Gene Ontology (GO) to annotate the sequences.

### Statistical analysis

Unless otherwise specified, all data were presented in mean ± standard deviation, with at least three samples per group observed. Statistical analysis was conducted using one-way or two-way analysis of variance. A p-value of less than 0.05 was considered statistically significant. Data were analyzed using GraphPad Prism 9.0 and SPSS 26.0.

## Results

### Moxibustion alleviates swelling and pain while ameliorating gait disturbances in mice with OA

**Fig. 1:**
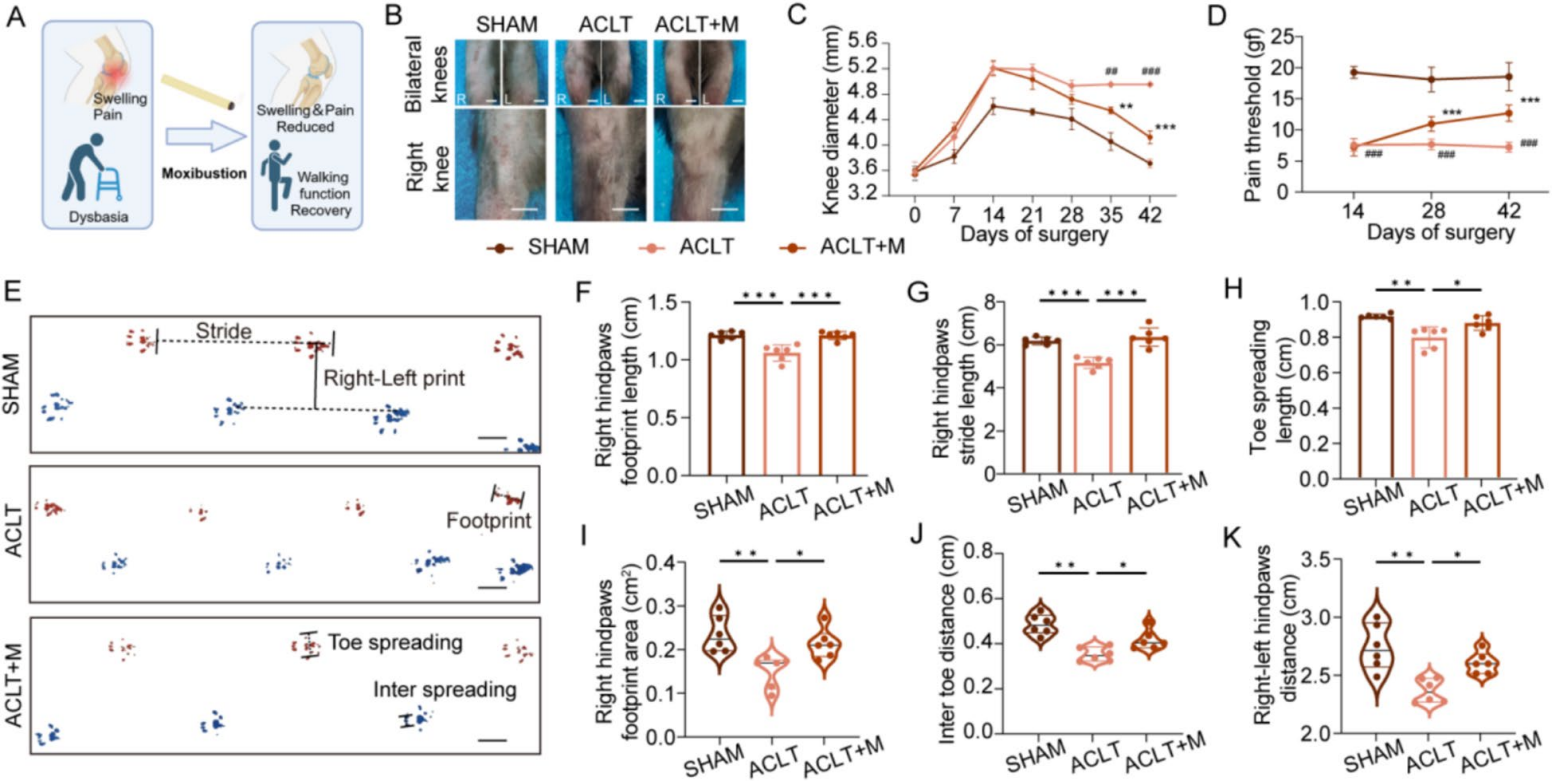
Moxibustion reduces swelling and pain and improves abnormal gait in knee osteoarthritis mice. **A** Schematic of Moxibustion’s effects on swelling reduction and pain relief. **B**, **C** Representative images and analysis of knee swelling degree in different groups. Scale bar: 2 mm (n = 6 per group). **D** The pain thresholds of mechanical-induced hyperalgesia in different groups (n = 6 per group). **E** Representative pictures of the footprints of each group. Scale bar: 1 cm. **F**–**K** Right hind paws footprint length, stride length, toe spreading length, footprint area, inter toe distance, and right-left hind paws distance of each group (n = 6 per group). Data presented as the mean ± SD.^##^*p* < 0.01, ^###^*p* < 0.001, vs. SHAM. *p < 0.05, **p < 0.01, ***p < 0.001, vs. ACLT

Clinical practice has shown that swelling and pain in OA are relieved by moxibustion intervention (Fig. 1A). We established an ACLT-induced OA model in C57BL/6J mice, and moxibustion was administered to mice for 28 days. During this period, knee joint diameters and mechanical pain thresholds were measured in each group to assess the effect of moxibustion on OA symptoms (Fig. S1A, B). The results showed that the diameter of the knee joints exhibited a notable increase during the initial 2-week postoperative period. However, by the third postoperative week, the joint swelling demonstrated a marked decline as the inflammatory response began to subside. The ACLT + M group showed a significant reduction in knee joint swelling during the third and fourth weeks of the moxibustion intervention, in comparison to the ACLT group (Fig. 1B, C). It turned out that on days 28 and 42 following the moxibustion intervention, the mean pain threshold of the right leg was considerably greater for the ACLT + M group than for the ACLT group (Fig. 1D). Furthermore, the ACLT + M group exhibited a notable increase in thigh circumference and limb strength in comparison to the ACLT group (Fig. S1D–F). Moxibustion had no significant effect on body weight changes in all groups of mice (Fig. S1C).

To assess the impact of moxibustion on the activity of OA mice, we collected footprints 4 weeks post-intervention for gait analysis (Fig. 1E and Fig. S1G), and a notable increase was observed in the right hind paw foot length, stride length, footprint area, right-left hind paw distance and toe spread in the ACLT + M group when compared with the ACLT group (Fig. 1F–K). These findings indicated that in ACLT-induced OA mice, moxibustion reduced knee joint swelling and pain and potentially improved abnormal gait.

### Moxibustion protects cartilage in ACLT-induced OA mice

**Fig. 2:**
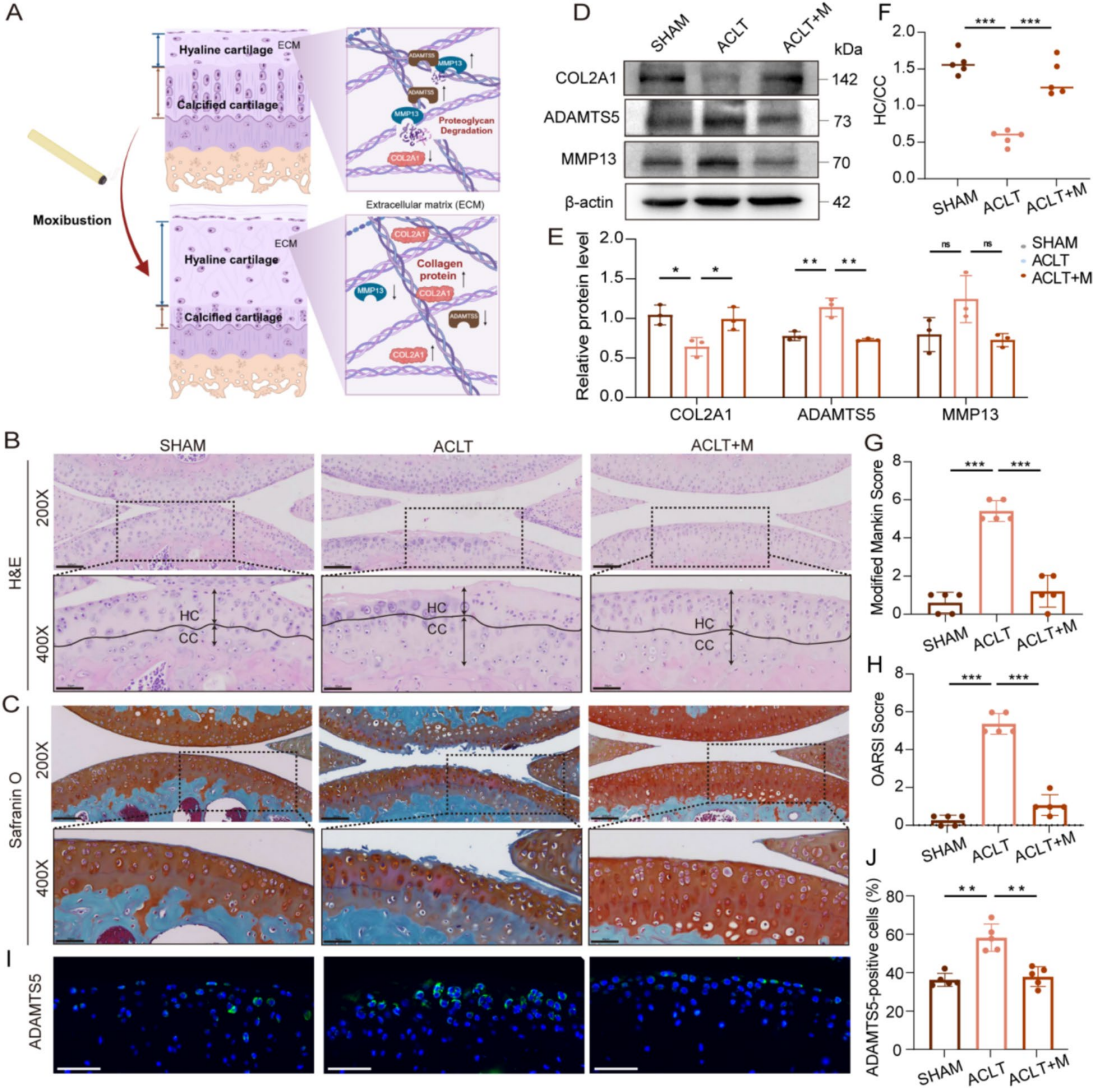
Protective effects of moxibustion on OA cartilage. **A** Schematic of moxibustion inhibiting ECM degradation in OA cartilage. **B** Representative images of H&E staining in knee joints. Scale bar: 100 μm; 50 μm. **C** Representative images of Safranin O-Fast Green staining in each group. Scale bar: 100 μm; 50 μm. **D**, **E** Western blot detection of the expression of COL2A1, ADAMTS5 and MMP13 proteins in the three groups (n = 3 per group). **F** Hyaline cartilage/calcified cartilage (HC/CC) in different groups (n = 5 per group). **G** Mankin score of B (n = 5 per group). **H** OARSI score of C (n = 5 per group). **I**, **J** ADAMTS5 positive expression in cartilage tissue (n = 5 per group). Scale bar: 50 μm. Data presented as the mean ± SD. ns, not significant, *p < 0.05, **p < 0.01, ***p < 0.001

It is well-established that degradation of cartilage extracellular matrix (ECM) leads to accelerated cartilage damage in OA (Fig. 2A). To evaluate the repair effect of moxibustion on cartilage damage, cartilage tissues obtained after moxibustion treatment were subjected to histological analysis using Safranin O-Fast Green and H&E staining. Our results demonstrated that, compared with the SHAM group, the hyaline cartilage of the knee joints in the ACLT group exhibited a notable reduction, while calcified cartilage demonstrated a substantial increase; additionally, the positive area of Safranin O was significantly diminished, indicating that the ECM had been impaired and the cartilage structure had degenerated (Fig. 2B, C). In contrast, moxibustion promoted the expression of collagen COL2A1 in the cartilage and decreased the levels of ECM-degrading enzymes ADAMTS5 and MMP13 (Fig. 2D, E, I, J). This delayed ECM degradation and apoptosis of chondrocytes (Fig. 2F–H), effectively mitigated the pathological alterations associated with OA.

### Moxibustion improves abnormal subchondral bone remodeling in OA

**Fig. 3:**
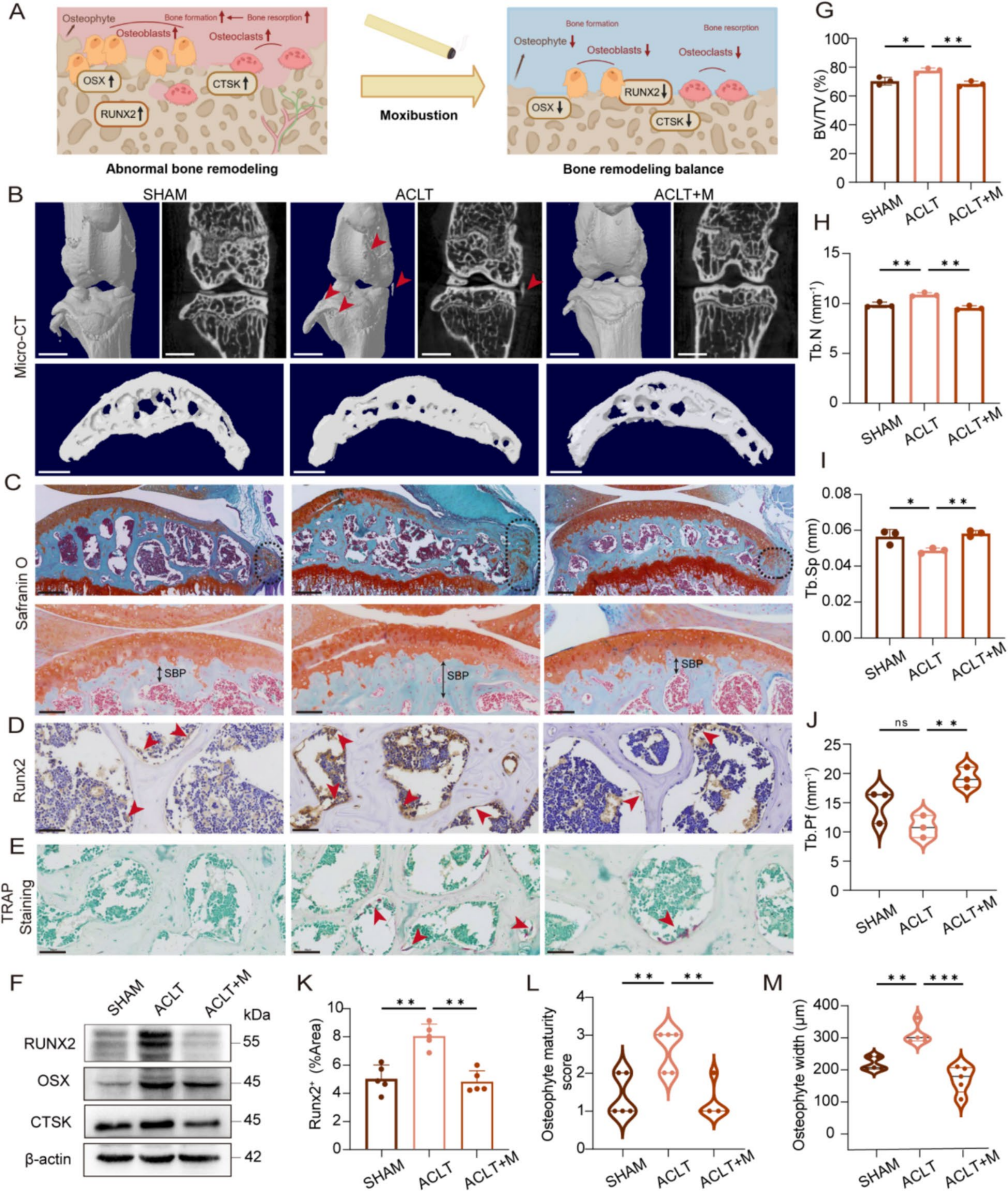
Moxibustion improves abnormal subchondral bone remodeling. **A** Schematic of moxibustion improving abnormal subchondral bone homeostasis in OA. **B** Representative images of subchondral bone by micro-CT. Coronal images show changes in the tibial and femoral surfaces and articular spaces, scale bar: 1 mm; sagittal images of the medial tibial subchondral bone show subchondral bone plate thickness and bone volume conditions, scale bar: 500 μm. **C** Representative images of sbchondral bone osteophytes and subchondral bone plates by Safranin O-Fast Green staining. Scale bar: 200 μm; 100 μm. **D** IHC images of RUNX2 protein level in subchondral bone tissues. **E** Representative TRAP staining images. **F** Western blotting detection of the expression of RUNX2, OSX, and CTSK proteins in subchondral bone. **G**–**J** Bone parameter analysis of subchondral bone: BV/TV, Tb.N, Tb.Sp and Tb.Pf (n = 3 per group). **K** Quantitative analysis of IHC staining of RUNX2 (n = 5 per group). (**L**, **M**), Semi-quantitative analysis of the width and maturity of osteophytes (n = 5 per group). Data presented as the mean ± SD. ns, not significant, *p < 0.05, ** *p* < 0.01, ***p < 0.001

It is believed that the disruption of bone remodeling homeostasis due to abnormal subchondral osteogenic and osteoclastic activity is an important pathological feature of OA and a contributing factor to articular cartilage degeneration [9, 32]. This study showed that moxibustion was effective in restoring the balance of subchondral bone homeostasis in OA (Fig. 3A). Three-dimensional reconstruction of knee joints by micro-computed tomography demonstrated that the increased thickness of osteophyte and the subchondral bone plate, particularly on the medial side, was exhibited in the ACLT group; additionally, there was a notable narrowing of the joint space, as well as heterogeneous bone formation on the  surfaces of the tibia and femur. These findings were improved by moxibustion and were consistent with the consequences of Safranin O-Fast Green staining (Fig. 3B, C). Moreover, the staining results demonstrated that, compared to the the bony osteophytes observed in the ACLT group, the subchondral bone osteophytes were predominantly cartilaginous structures and had a immature maturity in the ACLT + M group (Fig. 3L, M). Micro-computed tomography analysis of the subchondral bone region revealed deterioration of the subchondral bone microarchitecture in the ACLT group of mice. Although no considerable alterations between the ACLT group and the SHAM group were observed in the trabecular pattern factor (Tb.Pf), there was a notable increase in volume fraction (BV/TV) and trabecular number (Tb.N), accompanied by a significant diminution in trabecular separation (Tb.Sp) in the ACLT group. Moxibustion intervention significantly improved these changes and normalized the deteriorated structure (Fig. 3G–J).

The OA-related subchondral abnormalities were found to be alleviated following moxibustion treatment. Furthermore, the activities of osteoblasts and osteoclasts in the subchondral bone were assessed. The transcription factor RUNX2 serves a pivotal role in the differentiation of intercellular progenitor cells into osteoblasts. OSX, a downstream factor of RUNX2, is a crucial transcriptional activator in osteoblast differentiation [33]. Our findings revealed a significant elevation in the expression of osteogenic and osteoclastic differentiation proteins (RUNX2, OSX, CTSK) in the ACLT group compared to the SHAM group. It is noteworthy that the activity of osteoblasts was markedly more pronounced than that of osteoclasts in the ACLT group. However, the activity of both cell types exhibited a decline following the moxibustion treatment, with a more significant reduction in osteoblasts (Fig. 3D, F, K and Fig. S2D). In conclusion, moxibustion has the potential to improve the abnormal bone remodeling in OA.

### Moxibustion inhibits NLRP3 activation in OA subchondral bone

**Fig. 4:**
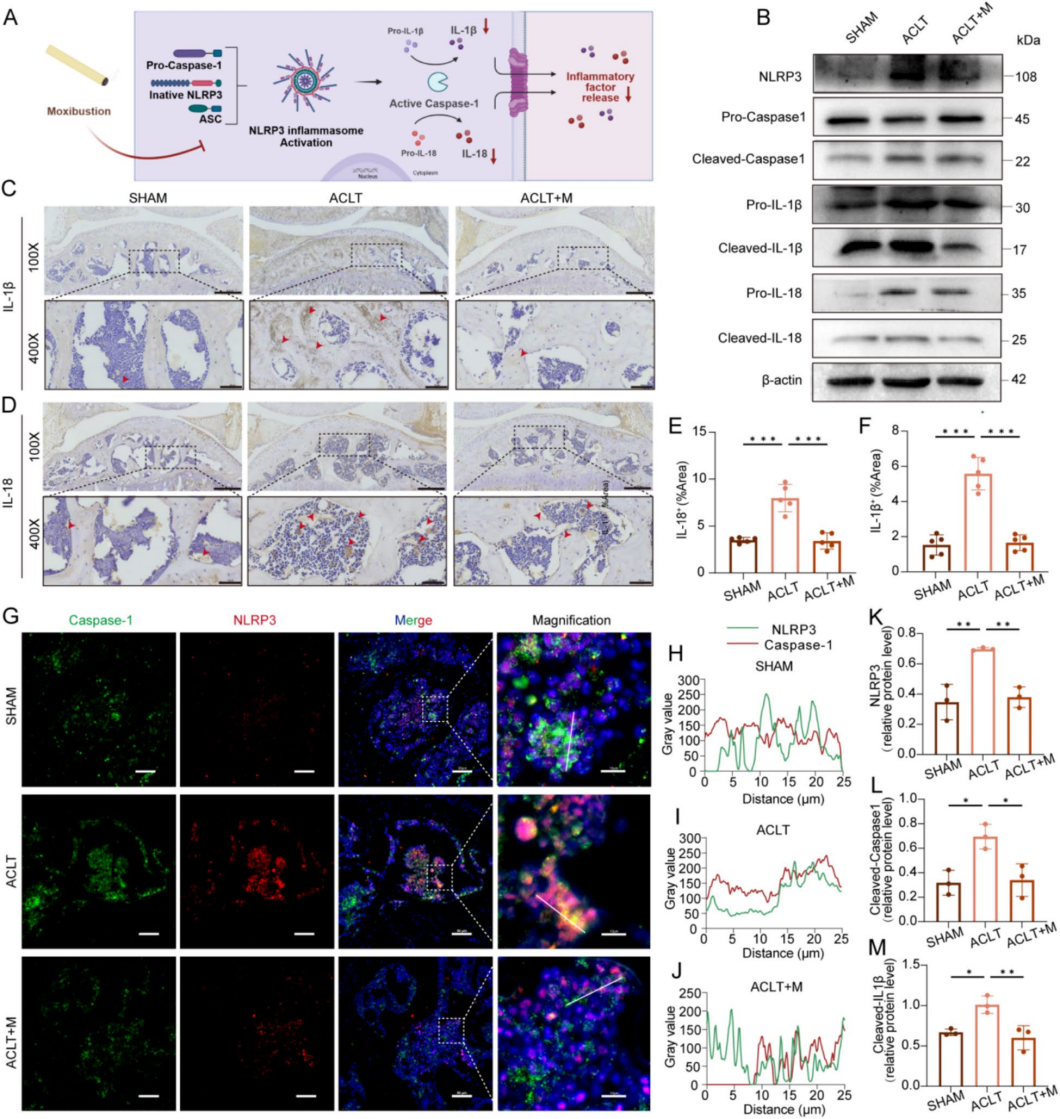
Moxibustion inhibits NLRP3 activation in OA subchondral bone. **A** Schematic of moxibustion inhibiting NLRP3 activation. **B** Western blotting detection of NLRP3, IL-1β, IL-18, and Caspase-1 proteins in subchondral bone. **C**, **D** IHC detection of IL-1β, IL-18 expression in each group. Scale bar: 250 μm; 50 μm. **E**, **F** Quantitative analysis of IHC staining of IL-1β, IL-18 in subchondral bone tissues (n = 5 per group). **G** Co-localization imaging of NLRP3 (red) and Caspase-1 (green). Scale bar: 50 μm; 10 μm. **H**–**J** Intensity of NLRP3 and Caspase-1 co-localization in different groups. **K**–**M** The expression levels of NLRP3, IL-1β, and Caspase-1 proteins in subchondral bone were detected by western blotting (n = 3 per group). Data presented as the mean ± SD. ns, not significant, *p < 0.05, **p < 0.01, ***p < 0.001

The NLRP3 inflammasome represents a crucial element of the inflammatory vesicle. Activation of NLRP3 has been demonstrated to induce inflammatory responses in tissues and cells, resulting in the production of pro-inflammatory cytokines such as IL-1β and IL-18 as well as degradative enzymes. Substantial evidence suggesting that NLRP3 is a pivotal player in the pathogenesis of a diverse range of arthritic diseases [34]. Activation of the NLRP3 inflammasome necessitates the exposure of pyrin domain (PYD) by NLRP3, which then allows for binding to apoptosis-associated speck-like protein containing a CARD (ASC). The cleavage and activation of Caspase-1 precursors recruited are the crucial steps in the inflammasome’s function (Fig. 4A). To further explore the mechanism by which moxibustion improves abnormal subchondral bone remodeling in OA, the inflammatory factors in the subchondral bone were initially examined. The results of immunohistochemical staining demonstrated  significantly increased expression of IL-18 and IL-1β in the ACLT group compared with the SHAM group. However, in comparison to the ACLT group, the expression of mature IL-1β and IL-18 as well as cleaved-Caspase 1 was markedly diminished in the subchondral bone of the ACLT + M group (Fig. 4B–F, M and Fig. S2C). The results indicated that moxibustion reduced the secretion of inflammatory factors and effectively reversed the inflammatory microenvironment. Further immunofluorescence co-localization and analysis displayed that the co-localization of NLRP3 and Caspase-1 in the subchondral bone was markedly elevated in the ACLT group relative to the SHAM group. This exhibited augmented intensity correlation, indicative of heightened NLRP3 inflammasome activation. Conversely, the expression and co-localization were diminished in the ACLT + M group (Fig. 4B, G–L and Fig. S2A, B). These findings indicated that moxibustion may restore subchondral bone homeostasis by impeding the assembly and activation of NLRP3 inflammasomes.

### Moxibustion-induced autophagy enhancement suppresses NLRP3 inflammasome activation

**Fig. 5:**
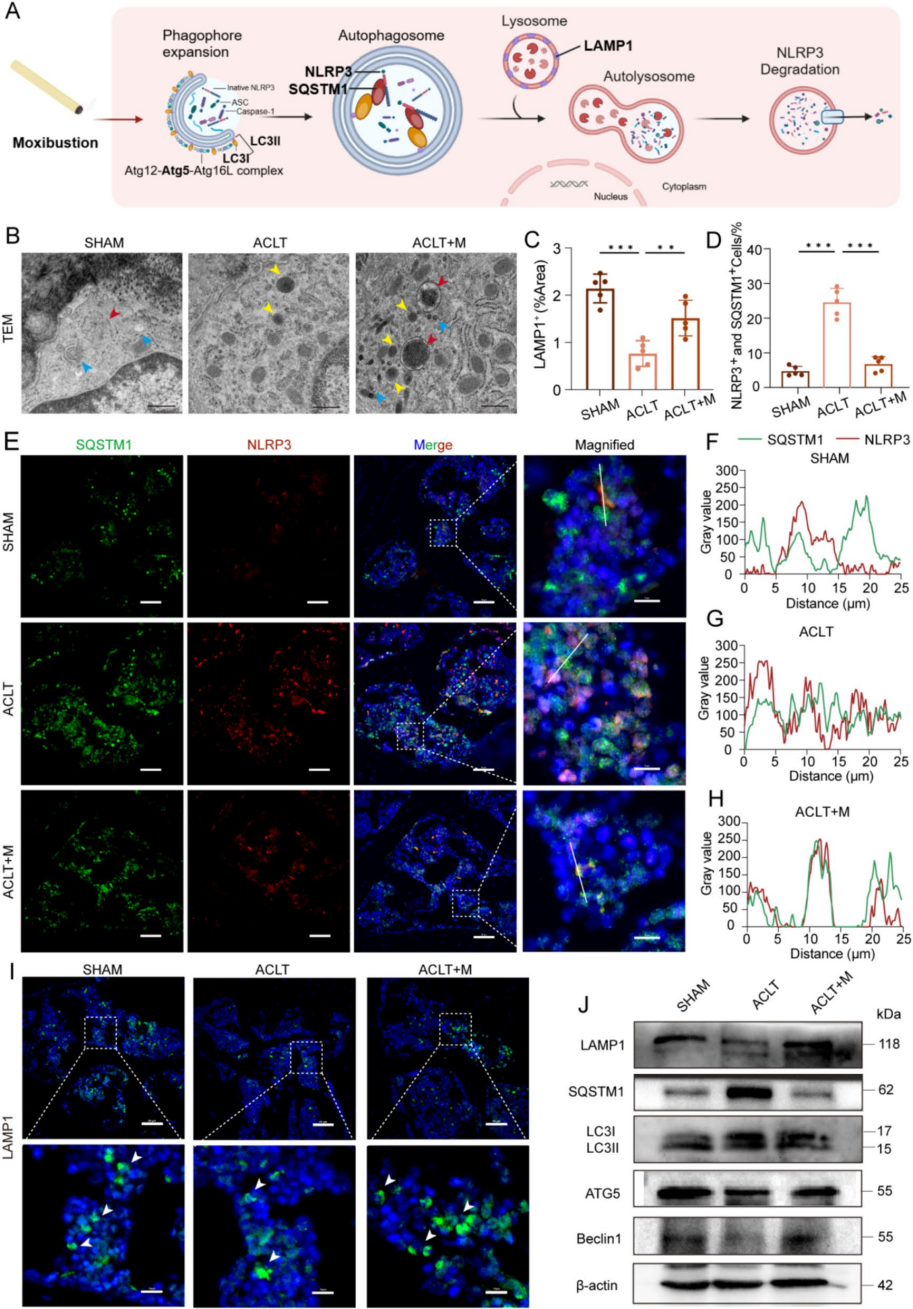
Moxibustion-induced autophagy and lysosome enhancement suppresses NLRP3 inflammasome activation. **A** Schematic of moxibustion promoting autophagy-lysosome pathway for NLRP3 degradation. **B** Representative images of autophagosomes (blue), lysosomes (yellow), and autolysosomes (red) in subchondral bone cells by TEM. Scale bar: 0.5 μm. **C**, **I** Analysis of LAMP1 immunofluorescence staining (n = 5 per group). Scale bar: 50 μm; 10 μm. **D**, **E** Co-localization images of SQSTM1 and NLRP3 in subchondral bone (n = 5 per group). Scale bar: 50 μm; 10 μm. **F**–**H** Intensity of SQSTM1 and NLRP3 co-localization in different groups. **J** Western blot detection of LAMP1, SQSTM1, LC3II/LC3II, ATG5 and Beclin1 proteins. Data presented as the mean ± SD. ns, not significant, *p < 0.05, **p < 0.01, ***p < 0.001

Furthermore, we investigated the alterations associated with NLRP3 inflammasome activity following moxibustion treatment. Numerous investigations have demonstrated that autophagosomes and lysosomes degrade the NLRP3 inflammasome, preventing its activation [18, 35]. Some studies have reported that moxibustion improves ultrastructure of articular cartilage by modulating NLRP3 [36], and our previous research has demonstrated that moxibustion prevents the release of inflammatory substances in OA by stimulating cartilage autophagy [27]. Therefore, we hypothesized that moxibustion promotes the autophagy–lysosome pathway to inhibit NLRP3 activation (Fig. 5A). We used transmission electron microscopy (TEM) to observe and analyze the autophagosomes, lysosomes, and autolysosomes in subchondral bone cells, and we discovered that in contrast to the other two groups, ACLT + M group exhibited a higher number of lysosomes and autolysosomes (Fig. 5B). Meanwhile, immunofluorescence analysis of NLRP3 and SQSTM1 (an autophagy substrate and autophagy regulatory protein) in subchondral bone demonstrated that moxibustion facilitated the co-localization of these two proteins, enhanced phagocytosis and promoted the degradation of NLRP3 via autophagosome, thereby inhibiting the NLRP3 activation (Fig. 5D–H and Fig. S2A).

Moreover, the ACLT + M group exhibited a markedly elevated level of positive LC3B, ATG5, and Beclin1 intensity in subchondral bone cells compared with the ACLT group (Fig. 5J and Fig. S3). This suggests that moxibustion treatment facilitates the formation of autophagosomes and restores autophagy–lysosome activity. Additionally, a reduction in LC3II/LC3I and an increase in SQSTM1 levels in the ACLT group of mice indicated decreasing autophagic flux in OA. Conversely, the ACLT + M group exhibited lower SQSTM1 levels and elevated LC3II/LC3I levels (Fig. 5J and Fig. S3G). The amount of LAMP1 at lysosomal membrane (Fig. 5C, I) increased in the ACLT + M group compared with ACLT group, indicating that moxibustion not only boosted autophagic flux but also improved lysosomal dysfunction in OA. Taken together, all these results collectively suggest that the activation of the NLRP3 inflammasome is inhibited by the autophagy–lysosome pathway.

### Moxibustion promotes ACSL1 expression in OA

**Fig. 6:**
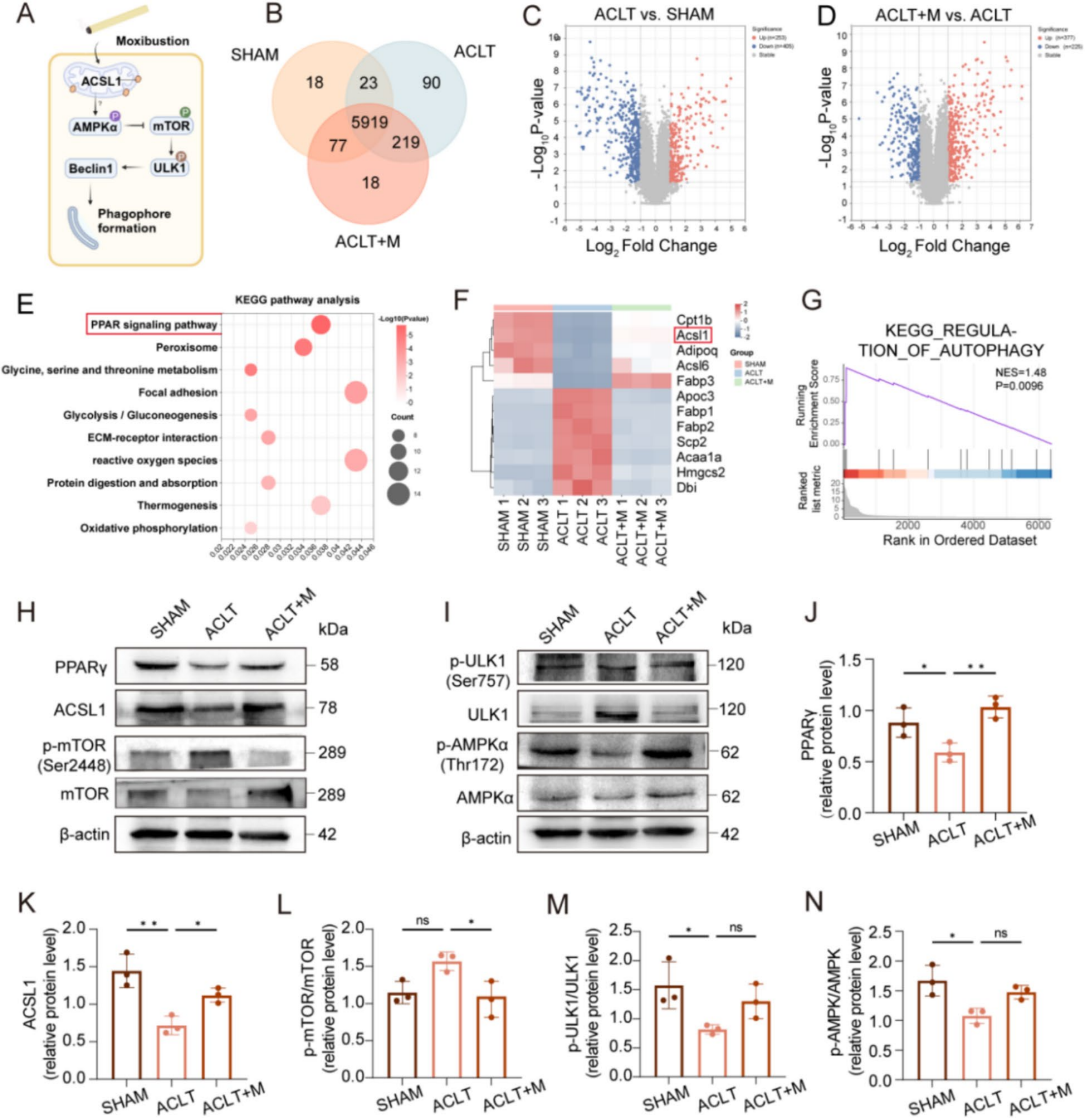
Moxibustion promotes ACSL1 expression in OA. **A** Schematic of moxibustion activating ACSL1 to promote autophagy pathway. **B** Venn diagram of protein expression in three groups. **C**, **D** Volcano plots of DEPS in the subchondral bone. **E** KEGG pathway analysis. **F** Heat map of DEPS in the PPAR signaling pathway. **G** Gene set enrichment analysis (GSEA) of regulatory pathways of autophagy between ACLT + M and ACLT groups. **H**–**N** PPARγ, ACLS1, P-AMPK/AMPK, P-mTOR/mTOR, and P-ULK1/ULK1 expression levels in different groups were detected by western blot (n = 3 per group). Data presented as the mean ± SD. ns, not significant, *p < 0.05, ** *p* < 0.01, ***p < 0.001

To elucidate the potential mechanisms by which moxibustion enhances autophagy to modulate subchondral bone homeostasis and combat OA, we performed proteomic sequencing using right knee subchondral bone from mice. There were 658DEPS (> 2- or < 0.5-fold; p < 0.05) in the ACLT group compared to the SHAM group, out of the 5919 total proteins identified (Fig. 6B). Among these, 253 proteins were up-regulated, and 405 were down-regulated (Fig. 6C). A total of 602 proteins exhibited differential expression (> 2- or < 0.5-fold; p < 0.05) between the ACLT + M and ACLT groups. with 377 up-regulated and 225 down-regulate (Fig. 6D). According to the Kyoto Encyclopedia of Genes and Genomes (KEGG) enrichment analysis of DEPS in the three groups, there was considerable enrichment in the peroxisome proliferator-activated receptor (PPAR) signaling pathway (Fig. 6E). PPARγ (PPAR can be further subdivided into three distinct isoforms: PPARα, PPARγ, and PPARβ/δ) deficiency has been reported to cause abnormalities in mTOR and autophagy signaling, exhibiting an accelerated OA phenotype [37, 38]. Consequently, we speculated that moxibustion might slow down OA progression by modulating the PPAR signaling pathway. The analysis showed that 12 DEPS were closely related to the PPAR pathway, five of which were up-regulated by moxibustion compared with the ACLT group (Fig. 6F). Notably, ACSL1, which is related with PPAR signaling pathway has been demonstrated to be closely associated with knee osteoarthritis [39] and NLRP3 [40]. In addition, gene set enrichment analysis (GSEA) showed that markedly higher enrichment scores of autophagy regulatory modules in the ACLT + M group compared with the ACLT group (Fig. 6G), which corroborated our experimental results.

The fatty acid oxidation rate-limiting enzyme ACSL1 is located in both the endoplasmic reticulum and mitochondria, where it plays a crucial role in maintaining cellular energy production. It has been reported that ACSL1 inhibits mTOR activation by triggering the energy sensor AMP-activated kinase (AMPK), thus increasing autophagy levels by promoting the phosphorylation of ULK1 [41, 42]. To further investigate the regulatory pathway of PPAR signaling on autophagy in subchondral bone, we hypothesized that moxibustion activates the AMPK–mTOR–ULK1 pathway via ACSL1, thus promoting autophagy (Fig. 6A). Our results showed a notable decline in the expression of both ACSL1 and PPARγ in the ACLT group compared to the SHAM group, which was markedly increased by moxibustion intervention (Fig. 6H, J, K and Fig. S4B, G). This observation suggests that moxibustion may activate PPAR signal and upregulate ACSL1 expression in OA subchondral bone. Our analysis of proteins related to the AMPK-mTOR-ULK1 pathway revealed that moxibustion promoted the phosphorylation of AMPKα and ULK1 in OA subchondral bone, and enhanced the level of energy metabolism and autophagy regulation.

## Discussion

Over the past 30 years, the number of osteoarthritis cases has increased by 132.2% [43], and the disease burden is projected to continue to rise all the way to 2050 [44]. As a safe, effective and low-cost treatment, moxibustion has been shown to be beneficial in alleviating knee osteoarthritis based on clinical studies and practice [23–25]; however, the specific mechanism of regulating the subchondral bone in OA is still unclear. Thus, in this research, we investigated the mechanism by which moxibustion improved subchondral bone remodeling in OA mice, and we found that moxibustion successfully reduced inflammation and alleviated abnormal remodeling in subchondral bone. According to our findings, moxibustion promotes autophagic flux and lysosomal degradation of NLRP3, relieving knee osteoarthritis pain and swelling. A series of experiments validated our identification of ACSL1, which regulates autophagy, as a crucial upstream signaling of moxibustion against OA based on proteomic data analysis.

One of the main reasons people seek medical treatment for OA is due to painful and swollen joints [45]; there is still a clinical need to lessen them. Localized pressure-related mechanical pain is an essential aspect of the pain in knee osteoarthritis, joint pressures in inflammatory or injured joints might be 10–18 mm Hg higher than those in healthy joints [46]. And the results of gait analysis showed that the footprints of OA were less organized and less distinct compared to those of the SHAM group. This could be because mice in the OA group had difficulty applying force properly due to pain or joint swelling, which limited knee movement and led to associated muscle atrophy and weakness, a condition that was relieved by moxibustion. Previous research has demonstrated that cellular inflammatory markers and deterioration of subchondral bone rich in blood vessels and nerves are both highly correlated with pain in patients with OA, in addition to joint effusion [9, 47].

Current understanding suggests that the pathophysiology of OA affects the entire joint, including the synovium, cartilage, and subchondral bone [2]. In response to acute or chronic stimuli, the microenvironment and bone remodeling homeostasis in subchondral bone are disturbed, which involves the decoupling of osteoclastic bone resorption and osteoblastic bone formation, resulting in the replacement of damaged bone with new bone, and the upregulation of proinflammatory cytokine expression [3, 8, 11]. It is widely accepted that bone resorption is enhanced in early knee osteoarthritis, and in late stages it manifests itself as enhanced bone formation leading to sclerosis of the subchondral bone as well as development of osteophytes and bone cysts [4, 48]. In this study, we constructed the OA model at 6 weeks of ACLT. The data showed that, compared to the SHAM group, osteoclast activity was elevated in the ACLT group, while osteoblast activity was significantly stronger. This led to an increase in the subchondral bone volume and trabeculae number, sclerosis of the subchondral bone and osteophyte formation. The findings indicate that possibly by the sixth week of ACLT, the increased activity of osteoblasts has taken a major role in subchondral bone reconstruction.

Increasing evidence suggests that acupuncture [49] and many compounds derived from natural herbs [50, 51] are effective in attenuating the deterioration of subchondral bone microstructure and OA progression by modulating subchondral bone cells activity. In this regard, our results showed that moxibustion was capable of ameliorating pathological subchondral bone remodeling in knee osteoarthritis by balancing activities of osteoblasts and osteoclasts. This study focuses on the subchondral bone, but whether  it is the only active or initial site where moxibustion exerts its therapeutic effects needs follow-up verification. Future studies should focus on tracking moxibustion-induced signals in real time in a mouse model to explore the response rate of the various tissues in OA under moxibustion intervention.

IL-1β and IL-18 pro-inflammatory cytokines are produced when NLRP3 is activated. These cytokines are believed to influence osteoblast and osteoclast differentiation, aggravating arthritic disease [11, 12]. Prior research has shown that OA cartilage and synovium exhibit markedly elevated NLRP3 expression [16, 52]. However, we unexpectedly found that NLRP3 inflammasome activity was also high in OA subchondral bone, and moxibustion effectively inhibited the NLRP3 activation and improved the inflammatory environment of subchondral bone. Bone reconstruction involves not only bone formation and resorption but also intricate interactions between immune cells and the osteogenic microenvironment, maintaining a dynamic balance through the secretion of cytokines that significantly regulate osteoclastogenesis and osteogenesis [3, 32]. We speculate that the inhibition of NLRP3 activation by moxibustion may occur in subchondral bone macrophages [53], osteoblasts [54] and vascular endothelial cells [55], which needs to be fully verified by further experiments.

We hypothesize that the combined action of the photothermal  radiation and volatile substances of moxibustion reduces inflammatory levels and improves blood circulation in the subchondral bone. Existing studies have shown that moxa thermal stimulation activates thermoreceptors such as TRPV, mediating local vasodilation, neuromodulation, and anti-inflammatory effects [56], whereas volatile oils have been shown to possess a wide range of biological activities such as anti-inflammatory, antioxidant, and immunomodulatory effects [57, 58]. With the current technical tools, it is challenging to accurately separate the  independent contributions of these two in vivo [59, 60]. Future research is therefore desperately needed to create experimental methods that can separate the different effects of moxibustion in order to fully examine the nature of moxibustion effects and optimize moxibustion therapy. The pure moxa-thermal, low/normal temperature moxa-volatile oil group and the conventional moxibustion group, for instance, could be directly compared; controlled thermal stimulation with different concentrations of volatile oil could be precisely applied, and the two could be treated separately or together to measure the individual and complementary biological effects.

In this research, we explored for the first time the potential therapeutic mechanism of moxibustion in the OA subchondral bone. Proteomic analysis revealed that ACSL1 is an important regulator in PPAR and reactive oxygen species (ROS) pathways (Fig. S4A). Several studies have shown that ACSL1 deficiency inhibits lipid metabolism and promotes glucose metabolism, thereby affecting AMP/ATP ratio and inhibiting AMPK activation [41, 42]. Our follow-up study verified that moxibustion upregulated the expression of PPARγ-indirectly-regulated ACSL1 expression in the subchondral bone, thereby protecting against OA progression. In addition, we found that the ROS pathway was prominently enriched in KEGG analysis. Our subsequent experimental results on Heme Oxygenase-1 (HO-1) indicated that moxibustion could improve the antioxidant level of OA subchondral bone (Fig. S4C–F). Studies have demonstrated that excessive ROS can activate the NLRP3 inflammasome [61]. Thus, the activation of NLRP3 inflammsome in OA subchondral bone may be related to overstimulation of ROS, in addition to autophagy insufficiency due to ACSL1 down-regulation, which requires further studies to elucidate their potential relationship.

 Moxibustion has garnered increasing attention in both research and clinical practice for managing osteoarthritis due to its noninvasive, relatively safe, and potentially efficacious effects, particularly in pain relief and functional improvement. This study clarified novel targets and underlying mechanisms of moxibustion treatment for OA. However, the following challenges remain: it is uncertain how ACSL1 interacts with other signaling pathways and whether moxibustion influences the subchondral bone through other mechanisms; the absence of  long-term evaluation of moxibustion's therapeutic effects on OA (e.g., more than 1 year); the lack of large-scale, high-quality, evidence-based medical studies on the optimal moxibustion regimen for different courses and types of OA; and the ambiguity surrounding how long moxibustion’s effectiveness will last. The intricate processes by which moxibustion reduces and regulates abnormal subchondral bone remodeling in OA can be better understood in the future through gene-edited animal models, superior clinical research, and pertinent in vitro studies. This will ultimately provide a safe, efficient, and long-lasting management option for patients with OA.

## Conclusion

**Fig. 7:**
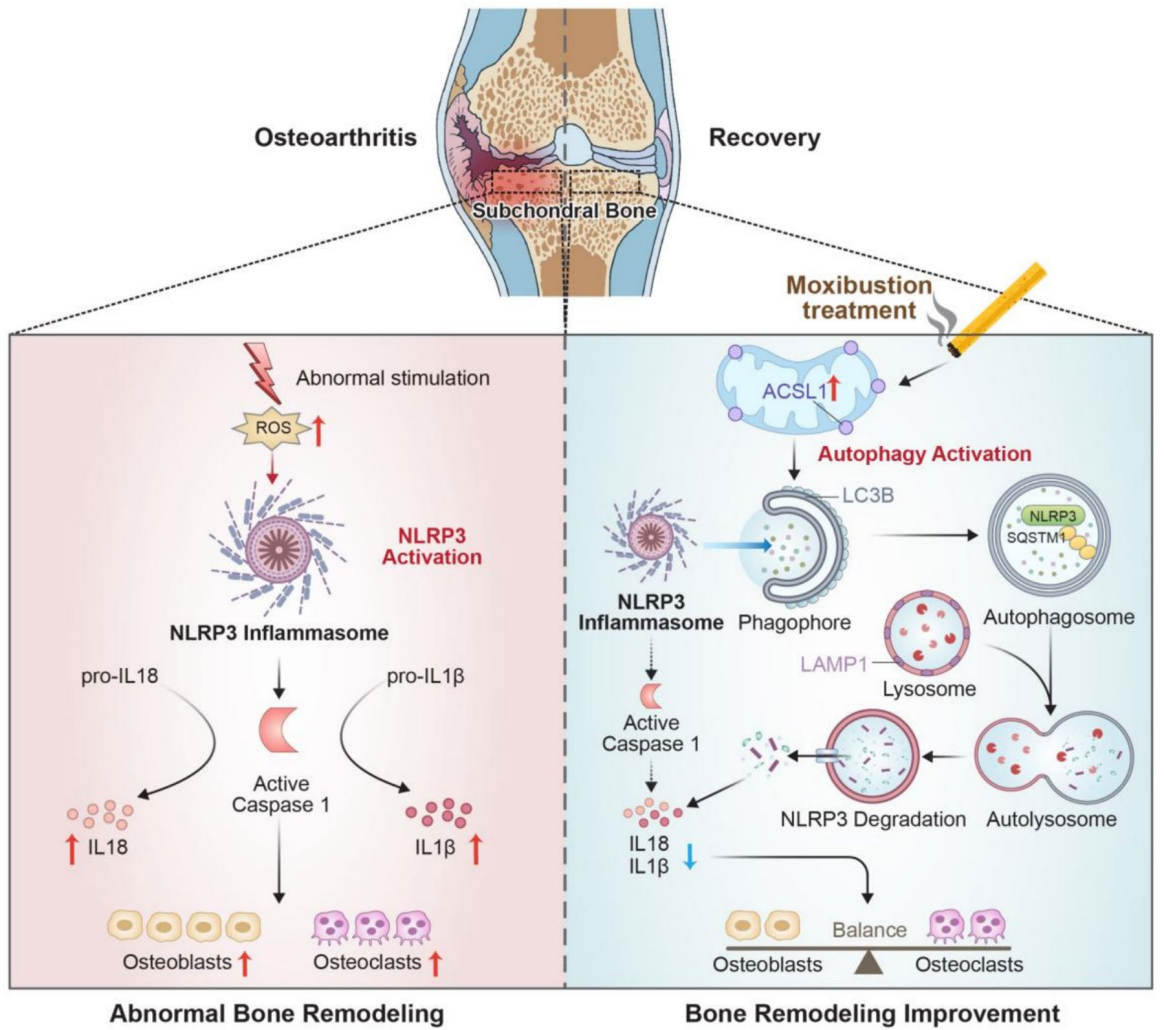
Schematic of the improvement of moxibustion against abnormal subchondral bone remodeling in OA. Abnormal stimulation triggers the activation of the NLRP3 inflammasome, leading to subchondral bone deterioration in OA. After moxibustion treatment, ACSL1 expression is upregulated, promoting increased autophagy levels to suppress NLRP3 activation, thereby balancing osteoclastic and osteogenic activities in OA. Ultimately, moxibustion treatment ameliorates abnormal subchondral bone remodeling and delays OA progression

For the first time, we investigated the specific mechanism by which moxibustion improves abnormal subchondral bone remodeling of OA mice (Fig. 7). The results demonstrated that moxibustion could activate the autophagy–lysosome pathway via ACSL1 up-regulation, promote the phagocytosis and degradation of NLRP3-related proteins, and thus reduce the secretion of inflammatory factors and maintain microenvironmental homeostasis. These findings provided a basis for future research into more comprehensive mechanisms of moxibustion in the treatment of OA and the development of more effective therapeutic strategies.

## Supplementary Information

Supplementary Material 1

## Data Availability

No datasets were generated or analysed during the current study.

## Abbreviations

ACLT: Anterior cruciate ligament transection
ACSL1: Acyl-CoA synthetase long-chain family member 1
ADAMTS5: A disintegrin and metalloproteinase with thrombospondin motifs 5
AMPKα: Adenosine 5ʹ-monophosphate (AMP)-activated protein kinase alpha
CTSK: Cathepsin K
H&E: Hematoxylin and eosin
IL: Interleukin
LAMP1: Lysosomal-associated membrane protein 1
LC3B: Microtubule-associated protein 1 light chain 3 beta
MMP13: Matrix metallopeptidase 13
mTOR: Mammalian target of rapamycin
NLRP3: NOD-like receptor thermal protein domain associated protein 3
OA: Osteoarthritis
OARSI: Osteoarthritis Research Society International
PPARγ: Peroxisome proliferator-activated receptor gamma
RUNX2: Runt-related transcription factor 2
TRAP: Tartrate resistant acid phosphatase
ULK1: UNC-51-like kinase 1
