# Supplementary material for: Moxibustion ameliorates abnormal subchondral bone remodeling by promoting ACSL1-mediated autophagy to degrade NLRP3 in osteoarthritis
Source: Chin Med. 2025 Aug 11;20:125. doi: 10.1186/s13020-025-01182-2 (PMC12337568; doi:10.1186/s13020-025-01182-2)
Supplement: Supplementary file 1 — Supplementary Material 1 [file 13020_2025_1182_MOESM1_ESM.pdf]

Supplementary materials

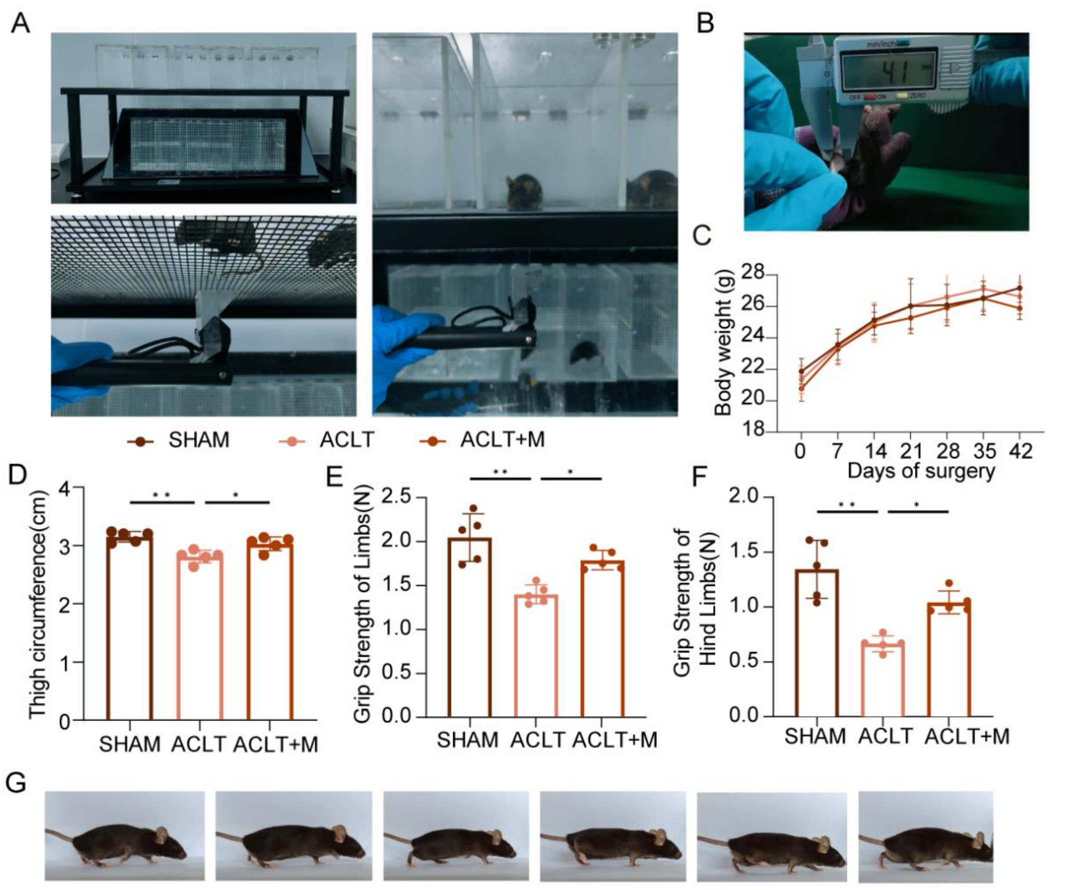

Fig. S1 Moxibustion effectively reduces swelling and pain, and strengthens muscles in OA mice, related Figure 1. **A** Measurement of right hind limb pain threshold. **B** Measurement of right knee joint diameter. **C** The body weight of the mice in different groups. **D** Thigh circumference of the right hind limb in each group (n = 5 per group). **E**, **F** Strength of limbs and hind limbs in each group (n = 5 per group). **G** Catching mouse footprints. Data presented as the mean±SD. *$p < 0.05$, ** $p < 0.01$, ***$p < 0.001$.

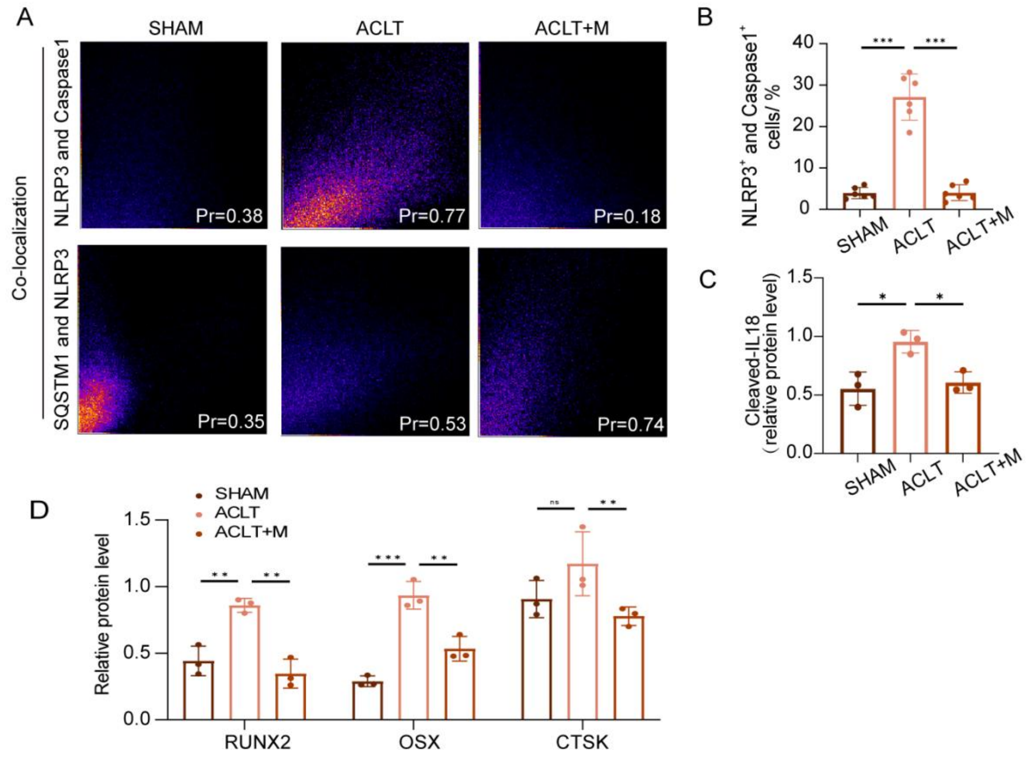

Fig. S2 Moxibustion-induced autophagy enhancement suppresses NLRP3 inflammasome activation, improving abnormal subchondral bone remodeling, related Figure 3-5. **A** Co-localization imaging and Parson correlation analysis of NLRP3 (red) and Caspase-1 (green), NLRP3 (red) and SQSTM1 (green). **B** Quantitative analysis of NLRP3 co-localization with Caspase-1(n = 6 per group). **C**, **D** The expression levels of Cleaved-IL-18, RUNX2, OSX, CTSK proteins in subchondral bone were detected by western blot (n = 3 per group). Data presented as the mean±SD. **p* < 0.05, ***p* < 0.01, ****p* < 0.001.

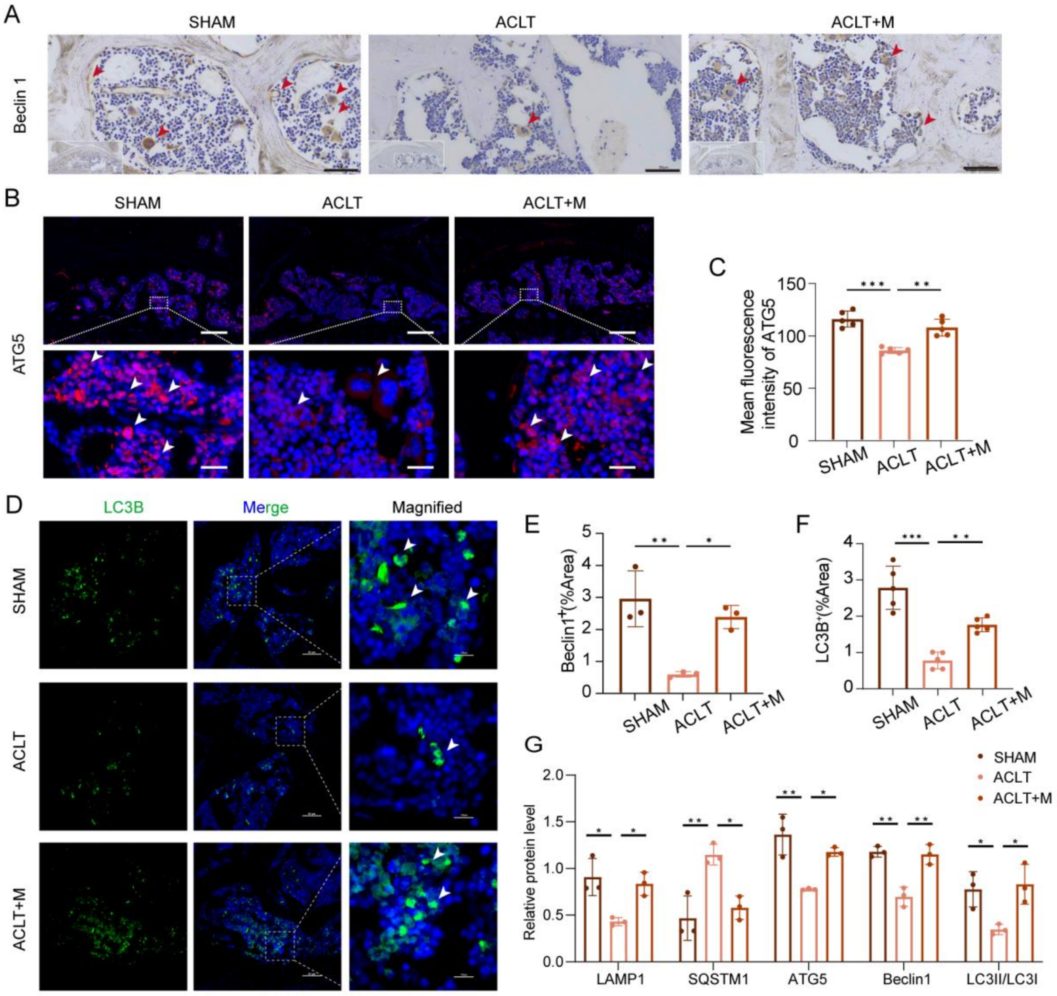

Fig. S3 Moxibustion promotes subchondral bone autophagy in OA, related Figure 5. **A**, **E** Analysis of Beclin1 IHC staining. Scale bar: 50 μm. **B**, **C** ATG5 positive expression in subchondral bone tissues of different groups (n = 5 per group). Scale bar: 200 μm; 20 μm. **D**, **F** Aanalysis of LC3B IF staining of different groups (n = 5 per group). Scale bar: 50 μm; 10 μm. **G** The expression levels of LAMP1, SQSTM1, LC3II/LC3II, ATG5 and Beclin1 proteins were detected by western blot (n = 3 per group). Data presented as the mean±SD. ns, not significant; *p < 0.05, **p < 0.01, ***p < 0.001.

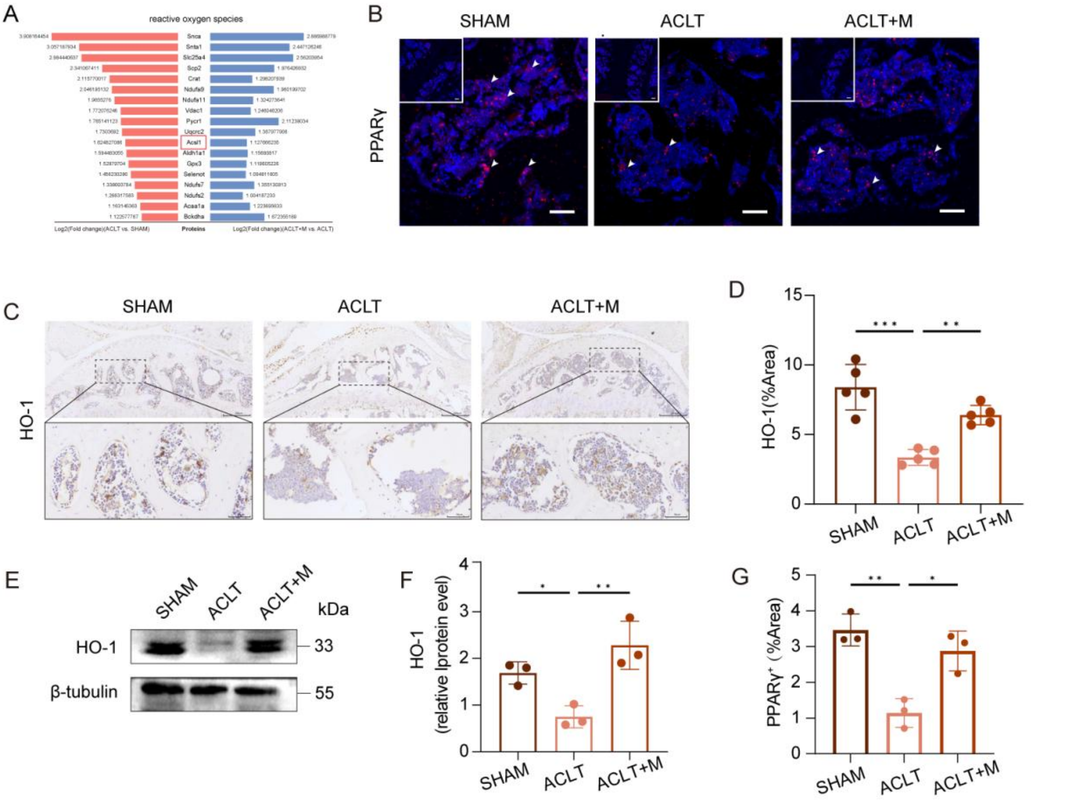

Fig. S4 Moxibustion promotes the expression of ACSL1 and PPARγ in subchondral bone of OA, related Figure 6. **A** Butterfly map of significantly differentially expressed proteins in the reactive oxygen pathway. **B**, **G** Analysis of PPARγ IF staining of different groups (n = 3 per group). Scale bar: 100 μm; 50 μm. **C, D** IHC detection and quantitative analysis of HO-1 expression in each group (n = 5 per group). Scale bar: 250 μm; 50 μm. **E, F** The expression levels of HO-1 proteins in subchondral bone were detected by western blotting (n = 3 per group). Data presented as the mean ±SD. *$p < 0.05$, **$p < 0.01$, ***$p < 0.001$.
